# Modeling TMJD pain in the laboratory mouse: role of TRP ion channels

**DOI:** 10.1186/1744-8069-10-S1-O8

**Published:** 2014-12-15

**Authors:** Yong Chen, Fan Wang, Wolfgang Liedtke

**Affiliations:** 1 Duke University Departments of Neurology, Anesthesiology and Neurobiology, Duke University Medical Center, Durham, NC 27710, USA

## 

Trigeminal pain syndromes such as temporomandibular joint (TMJ) pain appear to have a particular potential to affect patients in a devastating manner. Prevalence of trigeminal pain disorders in the US is estimated at 20-30x10^6^, at >50-75x10^6^ including headaches/migraine. Neural circuit malfunction and maladaptive plasticity arise from altered primary sensory afferents. We have focused on a nerve cell that is *the* eminent gatekeeper of sensory afferent cues in the trigeminal system, trigeminal ganglion (TG) sensory neurons. These neurons, when damaged by physical, inflammatory or chemical injury, set up the ensuing maladaptive reprogramming and circuit malfunction, including pathological pain, in the CNS.

In recent years, the importance of TRP ion channels, expressed in nociceptor neurons, has been recognized in pain transduction in response to physical and chemical-irritant cues [1,2]. TRP channels are non-selective cation channels with preference for Ca^2+^, so that nociceptor neurons can both be activated and reprogrammed [3,4]. One such candidate TRP channel with robust expression in TG sensory neurons is TRPV4 [5-7].

We have recently developed a novel method of bite force measurement in the laboratory mouse as a clinically relevant metric of TMJ that significantly extends current practice for assessing TMJ pain [8]. Taking advantage of this novel technique, our study shows that TRPV4 expression in TG sensory neurons plays a critical role in TMJ pain. Also, the expression of several other pain-related TRP channels and activation of extracellular signal-regulated protein kinase (ERK) in the TG after TMJ inflammation are regulated by TRPV4.

In addition, we have adopted the formalin irritant-pain model to trigeminally innervated territories in laboratory mice and examined the involvement of TRPV4. We found TRPV4 to be critically involved in trigeminal nocifensive behavior evoked by whiskerpad injections of formalin, a finding supported by studies in *Trpv4* null mice and with TRPV4-specific antagonists. Our results imply TRPV4 in MEK-ERK activation in TG sensory neurons, paralleling findings in chronic TMJ inflammation. Furthermore, cellular studies in primary TG neurons and in heterologous cellular systems with directed expression of TRPV4 suggest that TRPV4 can be activated directly by formalin to gate Ca^++^.

Taken together, these results imply TRPV4 as a critical signaling molecule in irritation─and TMJ chronic inflammation evoked trigeminal pain. TRPV4-antagonistic therapies can therefore be envisioned as novel analgesics for specific targeting of trigeminal pain disorders, such as migraine, headaches, TMJD, facial and dental pain, and irritation of trigeminally-innervated surface epithelia.

## Disclosures

Supported by NIDCR R01DE018549 (WL), R01DE19440 (FW), R01DE19440S1 (FW and WL), F33DE024668 (YC and WL); the Duke Institute for Brain Science (DIBS Incubator Award to FW and WL); and the Duke Nicholas School for the Environment (Leon Goldberg Fellowship to YC).
